# Current Aspects on the Plastic Nano- and Microparticles Toxicity in Zebrafish—Focus on the Correlation between Oxidative Stress Responses and Neurodevelopment

**DOI:** 10.3390/ani13111810

**Published:** 2023-05-30

**Authors:** Alexandra Savuca, Mircea Nicușor Nicoara, Alin Ciobica, Dragos Lucian Gorgan, Dorel Ureche, Ioana Miruna Balmus

**Affiliations:** 1 Doctoral School of Biology, Faculty of Biology, “Alexandru Ioan Cuza” University of Iasi, 700505 Iasi, Romania; alexandra.savuca@yahoo.com (A.S.);; 2 Doctoral School of Geosciences, Faculty of Geography and Geology, “Alexandru Ioan Cuza” University of Iasi, 700505 Iasi, Romania; 3 Department of Biology, Faculty of Biology, “Alexandru Ioan Cuza” University of Iasi, 700505 Iasi, Romania; 4 Academy of Romanian Scientists, No 54, Independence Street, Sector 5, 050094 Bucharest, Romania; 5 Center of Biomedical Research, Romanian Academy, 700506 Iasi, Romania; 6 Department of Biology, Ecology and Environmental Protection, Faculty of Sciences, University “Vasile Alecsandri” of Bacau, 600115 Bacau, Romania; 7 Department of Exact Sciences and Natural Sciences, Institute of Interdisciplinary Research, “Alexandru Ioan Cuza” University of Iasi, 700057 Iasi, Romania

**Keywords:** nano-/microplastics, oxidative stress, zebrafish, neurodevelopment

## Abstract

**Simple Summary:**

Recent reports focusing on the extent of plastic pollution have shown that petroleum-based products can currently be found in most marine species, which is a consequence of the immense production and use of plastics. The severe contamination of plastic nano-/microparticles (NPs/MPs) mainly results in immediate negative outcomes, such as organic impairments and tissue damage, as well as long-termed negative effects, such as developmental retardation and defects, chronic inflammation, oxidative stress (OS), metabolic imbalance, mutagenesis, and teratogenesis. We aimed to correlate the possible toxic effects of plastic NPs/MPs in zebrafish models, by focusing on OS and developmental processes, and the size, shape, and doses of the NPs/MPs. We found that plastic NPs/MPs toxic effects could be observed during the entire developmental span of zebrafish in close correlation with OS-related changes. The decreased antioxidant enzymatic defense due to plastic NPs/MPs exposure and accumulation suggests important neurodevelopmental negative outcomes (cognitive abnormalities, neurodevelopmental retardation, and behavioral impairments) and neuronal effects, such as impaired digestive physiology.

**Abstract:**

Recent reports focusing on the extent of plastic pollution have shown that many types of fibers and polymers can now be found in most marine species. The severe contamination of plastic nano-/microparticles (NPs/MPs) mainly results in immediate negative outcomes, such as organic impairments and tissue damage, as well as long-termed negative effects, such as developmental retardation and defects, chronic inflammation, oxidative stress (OS), metabolic imbalance, mutagenesis, and teratogenesis. Oxidative responses are currently considered the first line molecular signal to potential toxic stimuli exposure, as the oxidative balance in electron exchange and reactive oxygen species signaling provides efficient harmful stimuli processing. Abnormal signaling or dysregulated ROS metabolism—OS—could be an important source of cellular toxicity, the source of a vicious cycle of environmental and oxidative signaling-derived toxicity. As chemical environmental pollutants, plastic NPs/MPs can also be a cause of such toxicity. Thus, we aimed to correlate the possible toxic effects of plastic NPs/MPs in zebrafish models, by focusing on OS and developmental processes. We found that plastic NPs/MPs toxic effects could be observed during the entire developmental span of zebrafish in close correlation with OS-related changes. Excessive ROS production and decreased antioxidant enzymatic defense due to plastic NPs/MPs exposure and accumulation were frequently associated with acetylcholinesterase activity inhibition, suggesting important neurodevelopmental negative outcomes (cognitive abnormalities, neurodevelopmental retardation, behavioral impairments) and extraneuronal effects, such as impaired digestive physiology.

## 1. Introduction

Due to the immense production and use of plastics, residual and non-recyclable plastic nano-, micro-, and macroscopic particles have been found to accumulate in water, sea food, fish, and birds, eventually posing significant threat to human health [1,2,3,4]. Recent reports have shown that more than 1900 different plastic items (categorized by polymer composition) were found in seafood in the last year [5], while many fibers and polymer types were found in most marine species [6].

Considerable efforts have been made to describe the potential toxicity and mechanism of action of microscopic plastic particles, especially of polystyrene, mainly using animal models, such as rodents and fish. Thus, it has been shown that plastic nano-/microparticles (NPs/MPs) tended to accumulate in the lungs following inhalation from environmental sources [7], or in the gastrointestinal tract of some fish species from waters polluted with plastic NPs/MPs [8]. The severe intoxication of plastic NPs/MPs mainly resulted in immediate negative outcomes, such as organic impairments and tissue damage, as well as long-termed negative effects, such as developmental retardation and defects, chronic inflammation, OS, metabolic imbalance, and mutagenic and teratogenic potential [7,8,9,10,11].

In modern molecular toxicology, oxidative response is considered the first line molecular signal to toxic stimuli exposure [12]. While the modulatory activity of oxidative/reducing pathways and reactive oxygen species (ROS) signaling provides oxidative balance, the impairments that occur in processing and responding to harmful stimuli, as a result of abnormal signaling or dysregulated ROS metabolism, could be an important source of cellular toxicity [13,14]. In this context, OS could provide the molecular premise for the vicious stress cycle including both external (chemical, biological, and/or physiological) and oxidative signaling-derived toxicity [14,15,16]. As chemical environmental pollutants, plastic NPs/MPs are no exception to causing such toxicity, as a recent in vitro study on human brain cell lines demonstrated that exposure to polystyrene (PS) MPs is correlated with OS in a cause-effect relationship that is modulated by overproduction and accumulation of ROS [17].

By relation to oxidative response, plastic NPs/MPs toxicity has also been reported in several animal models, such as mice manifesting increased OS and inflammation in brain tissues [18,19], and mussels showing low enzymatic antioxidant defense in gills and digestive glands [20], and OS-mediated immunotoxicity [21], while fish exhibited severe OS in cerebral tissues [22]. PS, polypropylene (PP), polyethylene (PE), and poly (methyl methacrylate) (PMMA), solely or in mixture, have all been shown to cause similar toxic effects on oxidative metabolism and development [23,24,25]. Given our previous experience in zebrafish toxicology studies and the current interest in plastic NPs/MPs pollution, our aim was to describe, summarize, and correlate the possible toxic effects of plastic NPs/MPs at different stages in the life cycle using an animal model, focusing on the operating system and developmental processes. Efforts have also been made to hypothesize several possible OS markers useful in the assessment of plastic NPs/MPs poisoning.

Initially, zebrafish models were used in anticancer therapy research [26]. Once their genetic and phenotypical characterization was uncovered, their potential use for a wider range of research applications was outlined. Moreover, the physiological advantages of zebrafish contributed to their fast emergence in animal model research. The low breeding costs, simple maintaining conditions, and respective ethical regulations also contributed to their demonstrated versatility. Regarding the potential of zebrafish model in neurotoxicological and neurodevelopmental studies, Kalueff et al. [27] suggested that their value resided in the cognitive abilities, sleeping patterns, and brain tissues cellular morphology. Moreover, they described the similarities between the general macro-organization of the zebrafish and rodents’ brains, despite the increased evolutionary distance between the two animal classes. Recently, our group also thoroughly described these aspects and suggested that diverse measurable behavioral patterns are available to be assessed as early as pre-hatching stages of life [8,28]. Similarly, considering that the blood-brain barrier is functional from 36 hpf (hours post fertilization), zebrafish embryos could be used for neurotoxic effect evaluation for various molecules that are capable of penetrating the blood-brain barrier [29]. In this context, it is also valuable to mention that zebrafish reach the peak of cognitive development in 90 dpf (days after fertilization) through a process that progresses rapidly from the embryonic stage, larvae (72 hpf), juveniles (30 dpf), until the adult stage [30]. While behavioral and molecular models are gradually developing during this period, the range of biomarkers has becoming more complex and provides a powerful assessment tool for aggression, sleep, locomotion, memory, and social preferences [9,31].

The molecular patterns of oxidative balance develop concomitantly with the behavioral patterns. Despite the fact that the zebrafish brain is less complex than rodent and human brains, it is a major source of reactive oxygen species and is thus the most susceptible tissue to oxidative stress damage, one of the most common preserved traits. The effects of OS on the cerebral tissues and central nervous system were previously extensively discussed by our group while describing new pathophysiological patterns or bringing additional evidence to support the emergent OS pathological hypothesis in neurophysiology [9,32,33,34,35,36,37,38,39,40]. In this context, the evaluation of enzymatic and non-enzymatic antioxidants and OS effects on cellular and molecular structures could be observed in zebrafish in a tissue-specific manner but is not restricted to it [41].

Another advantageous feature of zebrafish is their completely sequenced genome and their facile manipulation by successfully using the innovative molecular techniques, such as the CRISP/Cas9 editing tool [42]. Moreover, 70% of the 26,206 zebrafish protein-coding genes have at least one orthologue in humans [43].

## 2. Plastic Nano-/Microparticles Accumulation and Oxidative Stress Response

**Figure 1:**
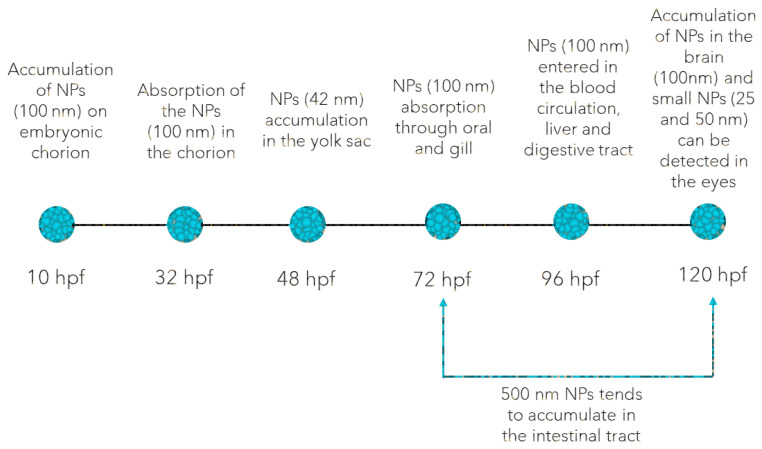
The possible target tissues of plastic NPs and MPs accumulation in zebrafish embryos and larvae [45,46,47,48,49,50,51,52,53,54,55,56].

Plastic MPs are solid masses of plastic that come from particles (size less than 5 mm) that consist of mixtures of polymers and various additives. These plastic MPs are either prefabricated or are derived from the gradual degradation of plastic materials in the environment. Plastic NPs (size less than 1 µm) exhibit a colloidal behavior which makes them more reactive and toxic than plastic MPs and their counterparts. This is mainly due to their very small size that facilitates their ingestion by animals and their penetration through biological membranes [44]. From a toxicological point of view, both plastic NPs and MPs have been reported to exhibit bio-accumulative behavior mainly in the soft tissues and to commonly predispose to OS disregarding the developmental stage [45]. For instance, PS NPs were found to accumulate in the brain, gills, blood, liver, and digestive tract of zebrafish immediately after hatching (Figure 1) and cause oxidative DNA damages and developmental malformations [46].

However, it has been shown that the chorionic barrier could prevent plastic MPs accumulation, when they are smaller than 100 nm. Duan et al. [47] recently described the accumulation dynamics of plastic NPs in zebrafish development. Starting with 72 hpf, plastic NPs were absorbed through oral intake and gills, while at 96 hpf, they were easily found in blood circulation, and gradually accumulated in the liver and digestive tract. Further bioaccumulation was observed at 120 hpf in the brain, eyes (<50 nm PS NPs), intestinal tract, and outer epidermis (>50 nm PS NPs) of the zebrafish larvae, as plastic NPs were able to penetrate the blood-brain barrier [47,48]. Similarly, Parenti et al. [49] reported that the PS NPs tended to accumulate in the intestinal tract and later migrate to other tissues in a size-dependent manner, as it was shown in 72 to 120 hpf embryos exposed for 48 h to 500 nm PS NPs. Furthermore, mild OS following short-term exposure to PS NPs of 500 nm has also been reported in zebrafish embryos: significantly increased SOD activity, decreased COX activity, but no relevant CAT and GPx activity changes [49]. Despite these, PS NPs have been shown to induce apoptosis in brain tissues of zebrafish embryos, developmental malformations, and excessive ROS activity [46].

The plastic MPs accumulation in zebrafish adults has been thoroughly described as variable, multifactorial, and size/shape dependent. While the smaller plastic MPs (i.e., 5 μm PS MPs) have been reported to mainly accumulate in gills, liver, and gut, the larger ones (i.e., 20 μm PS MPs) tend to accumulate only in gills and gut [50] following short-termed exposure (96 h). This could suggest that the circulatory system and the caliber of blood vessels plays an important role in the plastic MP transport though the body.

**Figure 2:**
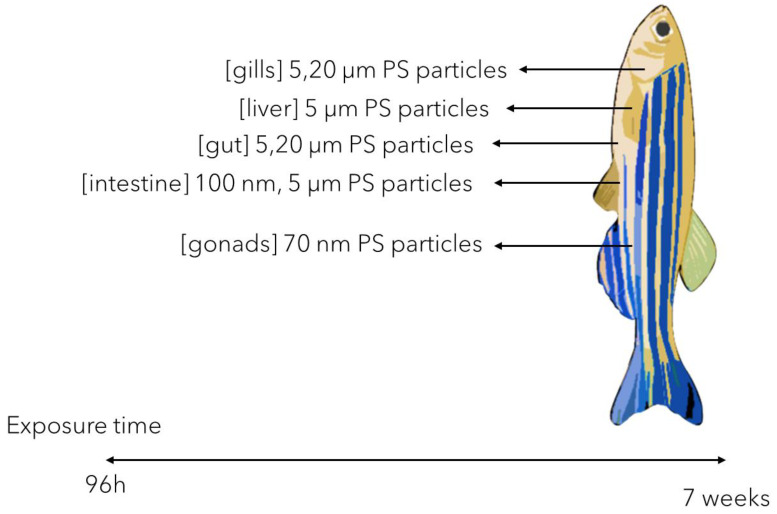
The possible target tissues of plastic NPs/MPs accumulation in zebrafish juveniles and adults [50,51,52,53,54,55,56,57,58,59,60,61,62].

Also, it has been reported that longer exposure times could further cause more severe molecular response in the tissues they accumulate in (Figure 2). Gu et al. [51] showed that both small (i.e., 100 nm) and larger (i.e., 5 μm) PS MPs accumulation in the intestines during a 21-day chronic exposure could lead to significant OS and sustained inflammatory response, as suggested by intense mucus secretion. Similar effects were also reported by Qiao et al. [52] showing, impaired intestinal lipid metabolism correlated with intestinal inflammation in a comparable exposure design (21 days exposure of zebrafish adults to 5 μm PS MPs). In this context, significant OS and tissue structure changes were reported consequently to liver antioxidant enzymes activity impairment (SOD and CAT) [50,52,53] (Figure 2).

Qiao et al. [53] observed that fibers, fragments, and beads tend to exhibit different accumulation patterns: plastic microfibers tend to cause more severe intestinal toxicity (mucosal damage, inflammation, microbiota’s dysbiosis, and intestinal metabolism disruption), as compared to micro-fragments and microbeads.

Furthermore, Pitt et al. [54] reported that the plastic NPs/MPs toxicity could extend beyond the individual effects, as they showed that plastic particles could be passed to offspring through maternal and paternal transfer. Plastic MPs accumulation in the chorion was described in F1 embryos, even if only one parent exhibited plastic particles intoxication. The accumulation in the yolk sac of the embryo was higher in co-parental transfer, as early as at 48 hpf for maternal transfer and at 72 hpf for paternal transfer. In this case, OS changes were reported in a sex-dependent manner. While muscular glutathione reductase (GR) was significantly decreased in both sexes, brain GR levels were significantly lower in females than in males, and conversely, in the gonads. Additionally, brain GPx activity was higher in the female zebrafish, while no significant changes were observed in CAT activity, as compared to male zebrafish [54] (as summarized in Table 1).

## 3. Plastic Nano/Microparticles Co-Exposure and Oxidative Stress Response

Considering the mixed exposure patterns that occur in the environmental conditions, extensive studies have shown that the interaction of plastic MPs with various environmental pollutants are needed to describe the correlations between co-exposure to multiple pollutants and their effects on development and molecular response. While many previous studies thoroughly described the effects of the main contaminants on development and OS, it was suggested that their interaction with other plastic NPs/MPs is based on aggregation, adsorption and transformation, which could lead to either synergistic or antagonistic effects, or harmful potentiating effects [63]. In this way, it has been reported that the 96 hpf co-exposure of plastic MPs with copper (Cu) could induce morphological malformations and abnormalities of the body structure, disruption of retina layers, and OS in embryos [64]. However, the decrease in SOD activity was dependent on Cu concentration, suggesting that MPs co-exposure could lead to potentiating or cumulative toxicological effect. Despite that, compensatory cellular responses to the increased ROS levels (increased GPx and CAT activities, and increased GSH levels) were observed following low and intermediate Cu concentrations and co-exposure. Thus, these results could suggest that following extensive exposure periods (14 dpf), plastic MPs could act as Cu vehicles, increasing the toxicological effects. All in all, plastic MPs solely or co-exposed with Cu could induce increased mortality rates, neurotoxicity (acetylcholine pathways modulation), and OS, in a suggestive synergistic manner (via bioavailability increase effect).

Similar synergistic effects were reported in short-term co-exposure (24 h) of PS nanobeads (50 nm) combined with gold (Au ions 1 μg/mL), which lead to an increased mortality rate, altered development, reduced hatching rate, and increased production of ROS in zebrafish larvae [65]. Regarding this aspect, Lee et al. [65] suggested that gold-induced ROS production was synergistically aggravated in the presence of PS nanobeads.

Long term exposure (three weeks) to PS microbeads combined with cadmium (Cd) in 18-weeks-old zebrafish adults led to increased plastic MPs bioaccumulation in the gills, liver, and gut [66]. Also, significant histological changes and plastic MPs concentration-dependent OS were reported in the tissues targeted by accumulation. GSH levels and SOD activity showed dose-dependent and co-exposure-dependent changes in the gills and gut. However, the most significant OS-related toxicological effects were observed for SOD activity in the gills of the Cd-treated fish, suggesting that plastic MPs could be implicated in Cd transport, but not in potentiating its toxicity. However, previous studies have shown that Cd is able to pass through the blood stream barrier and is predisposed to trigger ROS-mediated neurological impairments [67] without the assistance of plastic MPs.

Furthermore, it has been suggested that the toxicological effects of co-exposure to plastic MPs and other contaminants could further escalate when combined with other factors, such as exposure to ethylhexyl salicylate (EHS), one of the major organic UV filters commonly found in the environment. In this regard, Zhou et al. [68] reported significant OS changes (decreased SOD and CAT activities), suggesting that PS NPs could potentiate the pro-oxidative effects of EHS and its bioaccumulation in the offspring leading to mild OS. Subsequent increases in ROS and MDA content indicated that PS NPs could synergize the oxidative toxicity of EHS in offspring [68].

The co-exposure to various other contaminants has been shown to alter the antioxidant enzymatic defense and the oxidative balance. Increased MDA levels were found in adult zebrafish bodies following a 5-day exposure to PS NPs, metal oxides NPs, and polycyclic aromatic hydrocarbons, as compared to respective single treatments. On the other hand, decreased CAT activity was observed in both single and co-exposed groups, as compared to control. Similar effects were also reported in PS/Cu oxide or Zn oxide NPs and polycyclic aromatic hydrocarbons, in a potentiating manner [69] (as summarized in Table 2). This could suggest that the adsorption of toxic contaminants by plastic particles can alter the bioaccumulation and toxicity profile.

## 4. Behavioral Analysis and Oxidative Stress Response

Regarding the possible neurobehavioral effects of plastic NPs/MPs, recent studies have reported that PS NPs exposure alters larval behavior, as suggested by swimming hypoactivity in larvae [56] and impaired angle turning behavior in embryos [49]. Also, the latter study evaluated OS response following PS NPs exposure in embryos and reported significantly decreased SOD activity. Moreover, it has been shown that the negative effects of PS NPs exposure (similar types and sizes) could go even further, as Brun et al. [71] observed altered gene expression in glucose metabolism, cortisol secretion, and OS, correlated with impaired locomotor and behavioral patterns in zebrafish larvae, all of which could clearly suggest neurobehavioral toxicity. While the developmental neurotoxicity of plastic NPs was described, defective brain or nervous system functions in correlation with OS, such as increased CAT and GPx activity and ROS overproduction have been observed in zebrafish larvae [55].

However, the most frequent and constantly observed molecular response regarding OS following PS NPs intoxication was SOD activity impairment in all developmental stages. This pattern has been correlated with the reported developmental defects and behavioral impairments [49,50,52,53]. Regarding the possible molecular patterns of PS NPs toxicity, Pedersen et al. [56] described that the behavioral hyperactivity during dark cycles is correlated with dysregulated neurological and neuromuscular signaling and modulation pathways, generally occurring in neuropsychiatric disorders. For example, they reported impaired expression profiles of the *SLC6A1* gene, which is commonly found in ADHD models. Moreover, PS MPs lead to impaired locomotor activity (as suggested by the altered larval swimming behaviors observed in the free-swimming test) and to subsequent upregulated OS-related genes expression (i.e., CAT gene) [55].

Similar patterns were observed in zebrafish adults; Mak et al. [60] reported that abnormal behaviors, such as epileptic seizures, and erratic movement were observed following PE microbeads exposure. These impairments were generally correlated with genetic defects, illness, toxic agents’ exposure, or aging in adult individuals, while in larvae they were commonly associated with neurological phenotypes [72]. Moreover, Mak et al. [60] reported caudal fin deformation in adult zebrafish exposed to PE microbeads, which is regularly an important marker for morphoanatomical alterations that are suggestive of neurological seizures, tissue remodeling, pain, and/or inflammatory processes, all of which could indicate neurotoxic and neurodegenerative effects in adult individuals [73].

Taking into consideration that previous reports describing the significant correlations between affective disorders and OS [36], reduced aggressivity, impaired predator avoidance behavior, and social behavior associated with increased stress and anxiety has also reported in zebrafish adults following PS NPs exposure [62]. Furthermore, it has also been shown that PS NPs exposure resulted in impaired acetylcholine metabolism, mitochondrial chain, and excessive ROS production. Additional evidence on the correlation between the plastic MPs exposure and anxiety-like behavioral impairments was highlighted by Sarasamma et al. [62], who showed that the levels of some of molecules implicated in socio-affective behavior (oxytocin, vasopressin, serotonin, dopamine, and melatonin) modulation were impaired following PS NPs exposure.

In this way, the previously presented results could suggest that constant alterations in zebrafish behavioral patterns could be established as a result to plastic MPs exposure, and could be further correlated with several molecular changes occurring in brain signaling, toxic agents’ degradation, and OS. However, further studies regarding these aspects are needed, as the patterns were shown to be dependent on the type, size, and doses of plastic particles used in exposure. Also, the molecular mechanisms underlying the behavioral impairments and their correlation with OS mechanisms could be further studied in the context of plastic NPs/MPs intoxication, since the correlation between two out of the three components of this multifactorial interaction has already described [9,32,33,34,35,36,37,38,39,40].

## 5. Developmental Anomalies and Oxidative Stress Response

The developmental changes and impairments are among the most important evidences regarding the toxicological effects of chemical or environmental factors, with plastic NPs/MPs being no exception. In this way, the toxicological effects of plastic MPs effects on zebrafish development have been seen at every developmental stage. Recent studies have predominantly shown body malformations, such as pericardial and yolk sac oedema, abnormal tail, and axial curvature in zebrafish embryos [46,64]. Also, typical of toxicological evaluations, exposure to plastic MPs has been shown to induce increased mortality rates and delayed hatching, correlated with increased OS in the larval head region [47]. Furthermore, these changes suggested that the adsorption of PS NPs and MPs through the outer surface of chorion could lead to changes in chorion mechanical properties, further predisposing to embryonic hypoxic microenvironment. Regarding this aspect, Duan et al. [47] recently presented additional evidence that shows that the chorion property changes could be correlated with impaired heart rate, blood flow speed, and a slower hatching rate of the embryos, thus significant developmental toxicity.

The recent studies also showed that OS could be co-occurring with developmental defects as a result to NPs/MPs exposure. In this way, glutathione reductase (GR), GPx, and CAT activities have all been shown to be altered in developmentally impaired offspring, not necessarily in a sex-correlated manner [54]. Also, Chen et al. [58] reported both larvae body length reduction and increased CAT and GPx activities following PS NPs exposure. The correlation between the two different components was suggested by the changes occurring in the acetylcholine pathway impairments (as observed in the reduction of acetylcholine esterase activity). In this context, they suggested that the developmental abnormalities were significantly correlated with excessive ROS production, impaired CAT and GPx activity, and neurotoxic effects. As bradycardia was also observed in embryos originating from parents exposed to PS NPs [54], the developmental defects could also be the result of parental transfer.

A similar neurotoxic response was observed in the impaired swimming behavior in adults and in reduced larvae locomotor activity [49,56]. Different responses to stress and energy regulation networks, combined with hyperactivity, especially at night, could counteract potential motor dysfunctions and neurodegenerative effects to promote a hyperactive phenotype [57]. Antioxidant activity may be increased or inhibited under chemical stress depending on the intensity and duration of exposure.

Furthermore, the inhibition of acetylcholine esterase activity serves as a prominent biomarker of neurotoxicity since it causes severe neurotransmission impairment. As a consequence, the accumulation of acetylcholinesterase at synaptic gaps could induce hyperstimulation and ultimately death from respiratory or cardiac failure [74]. The sensitivity of acetylcholine esterase activity to various chemicals, including emerging pollutants in the environment such as nanomaterials, suggests the usefulness of this biomarker in providing an integrative measure of overall neurotoxic risk [75]. PS MPs could also cause gene expression modulated neurotoxic responses in the adult brain, including the inhibition of acetylcholine esterase activity in brain and liver, increased ROS activity, intense lipid peroxidation, and decreased CAT, SOD, and GPx activities in brain and liver [61].

## 6. Conclusions

The developmental and molecular toxic effects of plastic NPs/MPs depend on several factors, such as their size, shape, and doses. Each developmental stage can exhibit different patterns of behavioral and molecular responses following plastic NPs/MPs exposure. Developmental impairments, such as morphoanatomical defects and lower hatching rates, and molecular responses, such as upregulation of SOD, GPx, and CAT activity, have been commonly found in embryos exposed to plastic NPs/MPs. In zebrafish adults, the toxic effects of plastic NPs/MPs are similar and include significant OS (increase in ROS production, higher GPx, CAT activity), as well as behavioral and neurosignaling impairments (altered locomotion and socio-affective behavior correlated with OS and decreased AChE activity). Despite some variations in plastic NPs/MPs toxic effects throughout the individual development of zebrafish models, similar patterns of OS were generally seen. However, the SOD activity changes have often been reported to be dependent on the size of plastic NPs/MPs, suggesting that OS is relevant in the context of evaluating the molecular toxic effects of plastic NPs/MPs.

## Figures and Tables

**Table 1:** The effects of plastic NPs/MPs exposure in zebrafish models.

Life Stage	Plastic Characterization (Type/Shape/Size/Concentrations)	Accumulation Site	Exposure Time	Effects	Ref.
Embryo	PS/Beads/500 nm/1 mg/L	Intestinal tract	48 h (72 to 120 hpf)	↓ COX and ↑ SOD activities; Neurotoxic effects (changing their turning behavior).	[50]
	PS/20 nm/3 nL of 1% PS (injected)	Brain	120 h	↑ ROS production; Apoptosis, especially in the brain; Malformations in 60% of embryos; Mortality rate ~27%; Mild hatching delays.	[46]
Embryo/larvae	PS/Spherules/25, 50, 250, 700 nm/2, 25, 50 mg/L		120 hpf	25 and 50 nm PS NPs were found in the eyes; NPs > 50 nm were predominantly adsorbed through the intestinal tract and outer epidermis.	[48]
	PS/Spherules/100 nm, 157 ± 52 μm/250 MPs/50 mL; 2 × 10^4^ NPs/50 mL	Brain, gills, blood, liver, and digestive tract: - 72 hpf: PS NPs absorbed through oral and gill uptake; - 96 hpf: PS NPs entered the blood circulation, and gradually accumulated in the liver and digestive tract; - 120 hpf: PS NPs entered in the brain.	120 hpf	100 nm PS NPs can be blocked by the chorions; The adsorption of NPs/MPs by the outer surface of chorion can change the mechanical properties of chorion; NPs/MPs exposure induced altered heart rates and ↑ blood flow; ↓ Hatching rates of the embryos; NPs/MPs exposure can lead also to antioxidant system impairments in embryos.	[47]
	PS/Microspheres/1 µm/100, 1000 μg/L	Embryo—chorion (24 hpf) Larvae—mouths, stomachs, and intestinal tracts (96 hpf)	4–120 hpf	↓ Swimming ability of larvae; Inflammation and OS (significantly upregulated CAT gene expression).	[52]
Larvae	PS/50 nm, 45 μm	-	4–120 hpf	↓ AChE activity; ↑ CAT, and GPx activities; ↑ GSH levels; 6.1% ↓ body length; ↓ Locomotor activity ≥ significant developmental neurotoxicity.	[56]
	PS/50, 200 nm/2.46 × 10^14^; 2.54 × 10^12^ NPs/L	Gastrointestinal tract, liver, eyes, brain	6–120 hpf	Hyperactive behavior during dark cycles; Altered gene expression leading to neurological impairments, such as SLC6A1 (implicated in ADHD).	[55]
Embryo/larvae/ adult	PS/42 nm/5 mg/L	Embryo—chorion (maternal transfer, higher in co-parental transfer); Larvae—yolk sac (48 hpf maternal transfer, higher in co-parental transfer; after 72 hpf in parental transfer)	7 days	↓ GR activity: brain and muscles—females, muscles and testes—males; ↑ GPx activity in the female zebrafish brains; Bradycardia was observed in embryos from maternal and co-parental exposure groups.	[56]
Adult	PS/Beads/70 nm, 5 µm, 20 µm/20, 200, 2000 µg MPs/L	5 μm—gills, liver, gut; 20 μm—gills, gut.	7 days	↑ SOD and CAT activities in liver + impaired lipid and energy metabolism; 5 µm and 70 nm PS MPs: inflammation and lipids accumulation in liver.	[51]
	PS/Spherules/0.5 µm, 50 µm/100, 1000 μg/L		14 days	Intestinal microbiota alteration (PS MPs); Significant intestinal dysbiosis and inflammation (PS NPs, as compared to PS MPs).	[59]
	PS/100 nm, 5 µm, 200 µm/500 μg/L	100 nm and 5 μm—intestines	21 days	100 nm PS MPs altered the expression of phagocytosis-related genes; ↑ ROS and ↑ mucus secretion; Intestinal immune cells dysfunction; Pathogenic bacteria abundance.	[50]
	PS/Beads/5 µm/50 μg/L, 500 μg/L	Gut, liver, gills	21 days	↑ SOD and CAT activities (gut); Significant histological alterations; Significant alteration of lipid metabolism; Intestinal inflammation.	[52]
	PS/Beads/15 μm; PS/Fragments/4–40 μm; PP/Fibers/20–100 μm/10 μg/L	Gut (shape-dependent accumulation)	21 days	↑ SOD activity (gut).	[53]
	PE/Beads/10–22 μm, 45–53 μm, 90–106 μm, 212–250 μm, 500–600 μm/2 mg/L	89 ± 6% of intestine area	96 h	Erratic movements, epileptic seizures; Significant debilitating morphological alterations (abnormal tail bend); Increased ingestion disregarding beads size.	[60]
	PS/Beads/94–107 nm/10, 100 μg/L	Brain, liver	35 days	↑ ROS, ↑ lipid peroxidation; ↓ CAT, ↓ SOD, ↓ GPx activities (brain and liver); ↓ AChE activity (brain and liver); ↑ LDH and AST activities (brain and liver); ↑ ALT and ↓ AKP activities (liver); Histopathological damage: inflammation, degeneration, necrosis, and hemorrhage (brain and liver); Gene-modulated neurotoxic responses (brain). (dose and exposure time dependent changes)	[61]
	PS/70 nm/0.5, 1.5 mg/L	Gonads, intestine, liver, and brain	7 weeks	↓ Aggressivity and predator avoidance behavior; ↓ Social behavior (tight shoaling); ↑ Anxiety behavior; Hyperactivity in dark cycle; Lipid and energy metabolism impairments; ↓ AChE levels; ↓ oxytocin and vasopressin (brain); ↓ serotonin and dopamine activities; ↓ kisspeptin levels; ↓ melatonin; ↑ ROS, ↓ATP levels.	[62]

Abbreviations: PS—polystyrene; NPs—nanoparticles; MP—microparticles; COX—cyclooxygenase; SOD—superoxide dismutase; ROS—reactive oxygen species; CAT—catalase; AChE—acetylcholinesterase; GPx—glutathione peroxidase; GSH—reduced glutathione; ADHD—attention deficit hyperactivity disorder; GR—glutathione reductase; LDH—lactate dehydrogenase; AST—aspartate transferase; ALT—alanine transferase; AKP—alkaline phosphatase; ATP—adenosine triphosphate; ↑—increased; ↓—decreased.

**Table 2:** The effects of plastic NPs/MPs co-exposure in zebrafish models.

Life Stage	Experimental Conditions		Accumulation Site	Exposure Time	Effects	Ref.
	Plastic Characterization (Type/Shape/Size/Concentration)	Co-Exposed with				
Embryo/ Larvae	Polymers/Spherules/1–5 µm/2 mg MPs/L	60/125 μg/L Copper (Cu)	6 dpf: gastrointestinal tract	2 hpf—14 dpf	>10 dpf: ↑ Mortality; ↑ neurotoxicity (↓ AChE activity); ↑ ROS, ↑ SOD, ↑ CAT, ↑ GPx.	[70]
	Polymers/Spherules/1–5 µm/2 mg MPs/L	15/60/125 μg/L Copper (Cu)	Embryo—chorion, Larvae—yolk sac, brain, gastrointestinal tract	2–96 hpf	Morphological malformations (pericardial oedema, yolk sac oedema, spinal curvatures defects, retina layers disruption); ↓ Embryo survival and hatching rate ↑ Neurotoxicity (↓ AChE activity); ↑ ROS levels, ↓ lipid peroxidation, ↓ SOD, ↑ GPx activities (plastic MPs + Cu); dose-dependent CAT activity changes; ↑ GST and LDH activities (plastic MPs + Cu); ↑ GSH/GSSG levels (plastic MPs + Cu, Cu in higher concentrations).	[64]
	PS/Beads/50, 200, 500 nm/10 mg NPs/mL	1.25 μg/mL Au	Chorion, yolk sac; Lipid-rich regions (retina, brain).	24 h	↓ Survival, hatching rate; Developmental defects; ↑ ROS (co-exposure).	[65]
Adult	PS/Beads/5 µm/20, 200 μg MPs/L	100 mg/L Cadmium (Cd)	Gills, liver, gut	3 weeks	↑ GSH, ↓ SOD, ↑ MT activities; ↑ Inflammation. (PS MPs potentiated Cd accumulation)	[66]
	PS/Spherules/100 nm/10 μg NPs/L	1, 10, and 100 μg/L ethylhexyl salicylate (EHS)		28 days	↑ ROS and MDA, ↓ SOD activity; (PS NPs facilitated EHS transport and bioavailability)	[68]
	PS/Spherules/55 nm, 100 nm/1 mg NPs/L	nCuO (10 mg/L), nZnO (10 mg/L), chrysene (100 µg/L Chr), fluoranthene 100 µg/L (Flu)		5 days	↑ MDA and LPO, ↓ CAT activity (potentiating effect of co-exposure) (PS NPs more stable in PAHs mixtures, as compared with nMOx mixtures)	[69]

Abbreviations: PS—polystyrene; NPs—nanoparticles; MP—microparticles; AChE—acetylcholinesterase; GST—Glutathione-S-transferase; LDH—lactate dehydrogenase; GSH/GSSG—reduced/oxidized glutathione; MT—metallothionein; LPO—lipid peroxidation; nMOx—metal oxide; PAHs—polycyclic aromatic hydrocarbons; ↑—increased; ↓—decreased.

## Data Availability

The datasets used and analyzed during the current study are available from the corresponding author upon request.
